# Supplementary material for: The interactome of intact mitochondria by cross-linking mass spectrometry provides evidence for coexisting respiratory supercomplexes
Source: Mol Cell Proteomics. 2017 Dec 8;17(2):216–32. doi: 10.1074/mcp.RA117.000470 (PMC5795388; doi:10.1074/mcp.RA117.000470)
Supplement: Supplemental Data [file supp_RA117.000470_133922_0_supp_23978_fzffwf.zip › spectra_annotation/mito_DR_spectra_annotation/328-1-5-1-7-1.pdf]

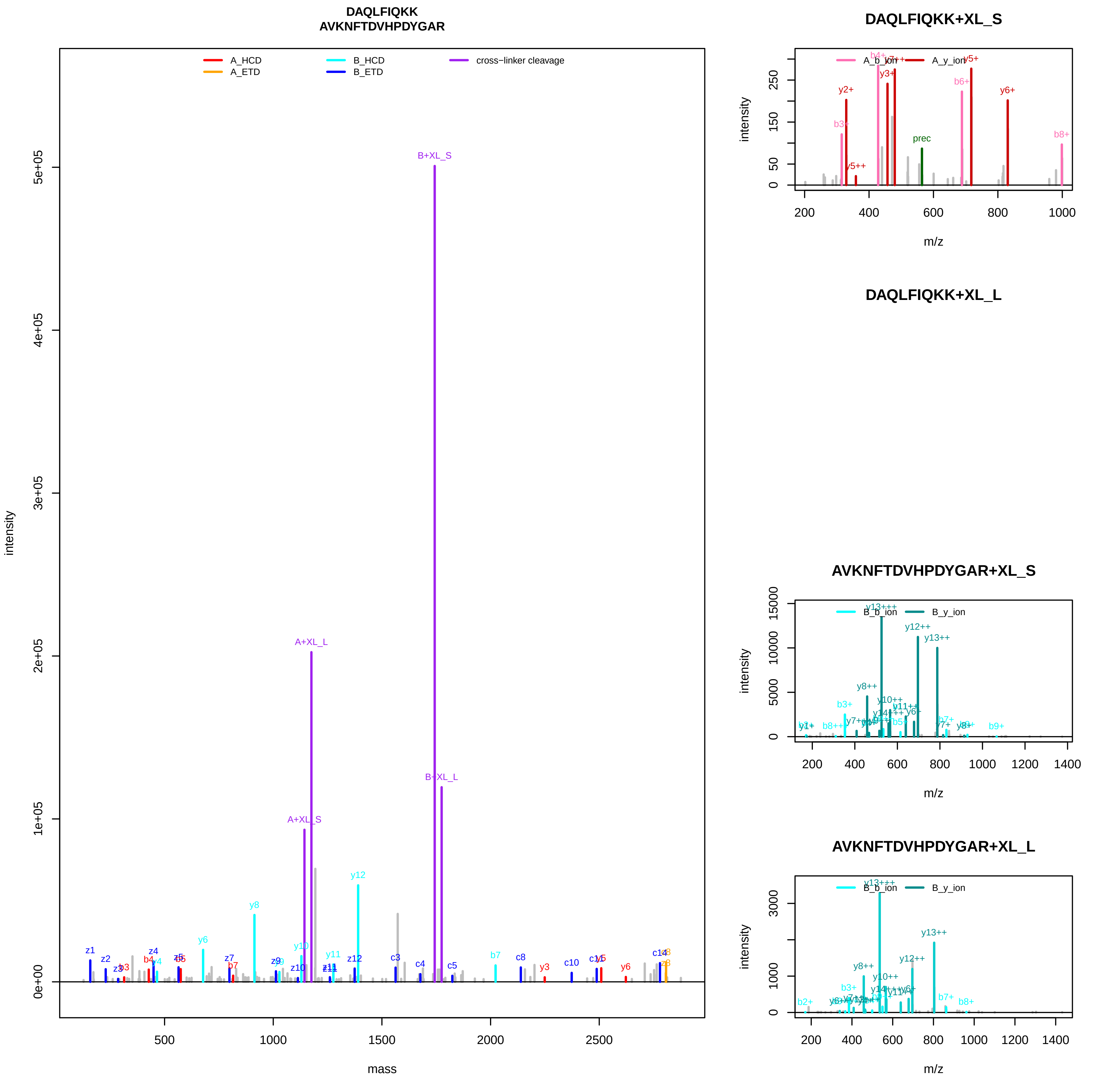
DAQLFIQKK
AVKNFTDVHPDYGAR
A_HCD
A_ETD
B_HCD
B_ETD
cross−linker cleavage
intensity
mass
0e+00
1e+05
2e+05
3e+05
4e+05
5e+05
500
1000
1500
2000
2500
B+XL_S
A+XL_L
B+XL_L
A+XL_S
y12
y8
y6
z1
z2
z3
z4
z5
z7
z9
z10
z12
c3
c4
c5
c8
c10
c14
b4
b7
b7
y3
y6
y11
y10
DAQLFIQKK+XL_S
A_b_ion
A_y_ion
intensity
m/z
y2+
y3+
y5+
y6+
b3+
b6+
b8+
y5++
prec
200
400
600
800
1000
0
50
150
250
DAQLFIQKK+XL_L
AVKNFTDVHPDYGAR+XL_S
B_b_ion
B_y_ion
intensity
m/z
y12++
y13++
y8++
y10++
b3+
b8++
b9+
0
5000
10000
15000
200
400
600
800
1000
1200
1400
AVKNFTDVHPDYGAR+XL_L
B_b_ion
B_y_ion
intensity
m/z
y13++
y12++
y8++
y10++
b3+
b2+
b7+
b8+
0
1000
3000
200
400
600
800
1000
1200
1400
